# Identification of novel mutant *PAX6 *alleles in Indian cases of familial aniridia

**DOI:** 10.1186/1471-2415-6-28

**Published:** 2006-06-27

**Authors:** Guruswamy Neethirajan, Jeyabalan Nallathambi, Subbaiah Ramasamy Krishnadas, Perumalsamy Vijayalakshmi, Shetty Shashikanth, Jon Martin Collinson, Periasamy Sundaresan

**Affiliations:** 1 Department of Genetics, Aravind Medical Research Foundation, Madurai, India; 2 Department of Glaucoma, Aravind Eye Hospital, Madurai, India; 3 Department of Pediatric Ophthalmology and Strabismus, Aravind Eye Hospital, Madurai, India; 4 School of Medical Sciences, University of Aberdeen, Aberdeen, UK

## Abstract

**Background:**

Haploinsufficiency at the *PAX6 *locus causes aniridia, a panocular eye condition characterized by iris hypoplasia and a variety of other anterior and posterior eye defects leading to poor vision. This study was performed to identify novel *PAX6 *mutations that lead to familial aniridia in Indian patients.

**Methods:**

Genomic DNA was isolated from affected individuals (clinically diagnosed aniridia) from nine unrelated aniridic pedigrees, unaffected family members, and unrelated normal controls. The coding regions of *PAX6 *were amplified and subjected to single strand conformation polymorphism (SSCP) gel analysis, and direct cloning and sequencing.

**Results:**

SSCP band shifts, indicative of DNA base pair mutations, were observed in five of these unrelated families. Four mutations were shown to be previously unreported insertion or deletions in *PAX6*, leading to frameshifts. These new mutations were c.1174delTG (in exon 10), c.710delC (exon 6), c.406delTT (exon 5) and c.393insTCAGC (exon 5). The other nonsense mutation, a transition (c.1080C>T) in exon 9, has been reported previously as a mutation hotspot for *PAX6 *in other ethnic pedigrees. All mutant alleles transmitted through aniridic individuals in each family.

**Conclusion:**

These new deletions and an insertion create frameshifts, which are predicted to introduce premature termination codons into the *PAX6 *reading frame. The genetic alterations carried by affected individuals are predicted to lead to loss-of-function mutations that would segregate in an autosomal dominant manner to subsequent generations. This is the first report of the 'hotspot' c.1080C>T transition from Indian families.

## Background

Congenital absence of the iris is known as aniridia in humans. Aniridia is mostly a panocular defect leading to anterior and posterior segment defects that are associated with isolated cataracts, macular hypoplasia, keratitis, and Peter's anomaly [1]. One third of cases are sporadic and two thirds are familial with an autosomal dominant mode of inheritance with high penetrance [2]. *PAX6*, from chromosome band 11p13, was identified as the candidate gene for aniridia by positional cloning [3]. *PAX6 *is widely expressed in the developing eye and is one of the master control genes in eye development. Molecular genetics studies have revealed that autosomal dominant aniridia results from many possible mutations in the *PAX6 *gene [4-7]. The *PAX6 *gene spans 22 kb and consists of 14 exons. The translation initiation codon is in exon 4 and the termination codon is in exon 13 [4]. The human *PAX6 *gene has paired box and homeobox motifs that encode a highly conserved protein of 422 amino acids among metazoans. The paired domain and the homeodomain are DNA binding domains, and are separated by a linker segment (LNK) and followed by a C-terminal region rich in proline, serine and threonine (PST). *PAX6 *functions as a transcription factor to regulate the expression of other genes during embryogenesis and in the adult eye [8]. Mutations in the *PAX6 *have so for been described in sporadic cases from India [6,7], but there was no previous report on mutations in Indian familial aniridia. We therefore present an analysis of five Indian pedigrees out of nine with hereditary aniridia demonstrating four novel mutations and one previously reported nonsense genetic alteration of *PAX6*.

## Methods

### Clinical evaluation and proband selection

The study protocol adhered to the tenets of the Declaration of Helsinki. After providing informed consent, nine unrelated clinically diagnosed inherited aniridia families (ANF1 to ANF9) with their available unaffected members and 60 healthy non-aniridic unrelated normal controls were recruited for this study. Ophthalmic specialists selected these families in the first instance because of iris absence, and classical aniridia was confirmed by the presence of secondary eye defects consistent with aniridia, and no contraindications. Examination included slit lamp, gonioscopy, intraocular pressure measurement, and biomicroscopy. Peripheral blood samples were collected for isolation of total genomic DNA by salt precipitation from probands, affected, unaffected family members, and normal controls.

### Mutation screening and sequence analysis

Exons 4–13 of the human *PAX6 *gene were amplified using previously described primers [4] in a 20 μl reaction mixture containing 100 ng genomic DNA, 100 pM of each primer, 1X PCR buffer (Promega, USA) and 0.5 U of Taq DNA polymerase (Promega, USA). PCRs were carried out in a MJ Research thermocycler for 35 cycles. PCR conditions were 1 minute at 95°C for denaturation, 1 minute at 58°C for primer annealing and 10 minutes at 72°C for primer extension. The amplified PCR products showing a single band of the correct size were stored at -20°C before electrophoretic analysis. For single-strand conformation polymorphism (SSCP) analysis, approximately 6 μl of the amplified DNA was mixed with 4 μl of denaturing dye (95% formamide, 10 mM NaOH) and run on a 10% polyacrylamide gel with or without 2.5% glycerol. Electrophoresis was carried out for 6 hours at 800 V. The gels were stained with silver nitrate as described previously [6]. Based on the mobility shifts seen on the SSCP gel, exons containing potential mutations were re-amplified and the PCR products were purified by excising the desired band from the agarose gel and eluting using a QIA quick gel extraction kit (QIAGEN Gmbh, Germany). PCR products were either sequenced directly or cloned into pGEM-T (Promega USA). The superimposed mutant PCR products were amplified, ligated, then transformed into *E. coli *DH5α and plated onto LB agar medium containing ampicillin. Transformed colonies were patched on a different plate for the isolation of plasmids. About 10 individual colonies were picked from each ligation and then plasmids were isolated by alkaline lysis. These plasmids were checked by restriction enzyme digestion and used as templates in PCR reactions for amplification of the insert. The mutants were detected by allele specific clones

The PCR products were sequenced on Applied Biosystems (ABI) model 3730 automated sequencer (Microsynth, Switzerland) using PCR primers from published literature [4] or universal primers for pDrive (QIAGEN, Germany).

### Digestion

Ten microliters of the mutant and normal PCR products were digested in a reaction mixture containing 1X buffer and 10 U of appropriate restriction enzyme (Fermentas, Germany). The reaction was incubated at 37°C for 2 hours and the products were visualized on a 2.5% agarose gel.

### *PAX6 *cDNA numbering

*PAX6 *cDNA sequences used and mutations were annotated according to the PAX6 Allelic Variant Database [9]. The coding region runs from base 363 (in exon 4) to base 1628 (in exon 13). The PST region extends from base 1169 to base 1628.

## Results

We screened for *PAX6 *mutations in genomic DNA from nine Indian pedigrees with familial aniridia in which more than one person was affected. The probands recruited in this study have typical bilateral aniridia with primary complications. The clinical information on the affected members is given in Table 1. Exons 4–13 of *PAX6 *(encompassing the entire coding region) were amplified from affected and unaffected individuals, as well as unrelated unaffected controls. PCR products were used for single-strand conformation polymorphism (SSCP) analysis. In five out of nine families, the affected members showed unusual band shifts on the 10% polyacrylamide gel. These shifts are caused by mono-allelic base sequence changes. Mutant alleles identified by SSCP were cloned and sequenced to determine the nucleotide changes underlying the aniridic phenotype. All mutant alleles are summarised in Table 2.

**Table 1:** Clinical information of familial aniridia patients

Patient	Best Vision		Refractive Error		Nystagmus	Keratopathy		Iris	Cataract		Glaucoma		Fovea Hypoplasia		Macular Hypoplasia		Treatment			
Number [Sex] (Age)																	Medical treatment		Surgical treatment	
																	
	R	L	R	L		R	L		R	L	R	L	R	L	R	L	R	L	R	L

ANF2-1 F (5)	CF	6/60	-	-5.0-2.0	+	+	-	R	+	-	+	+	+	+	-	-	+	+	1,2	1
ANF2-3 M (11)	PL	5/60	-	+10	+	+	-	R	-	+	-	+	+	+	-	-	-	+	-	1,2
ANF2-2 F (28)	PL	3/60	-2.0	-	+	+	-	A	-	-	-	-	+	+	-		-	-	-	-
ANF6-1 F (10)	4/60	6/36	-2.0	-	+	+	+	A	+	+	-	-	+	+	+	+	-	-	-	-
ANF6-2 F (24)	3/60	3/60	+11	+11.5	+	+	+	A	+	-	-	-	+	+	-	+	-	-	-	-
ANF7-1 M (0.5)	NA	NA	-	-	+	-	-	R	-	-	-	-	+	+	-	-	-	-	-	-
ANF7-2 F (22)	6/18	PL	+11	-	+	-	+	A	+	-	-	-	+	+	-	-	-	-	2	-
ANF9-1 F (5)	6/24	6/24	-4.0	-4.0	+	-	-	A	-	-	-	-	+	+	-	-	-	-	-	-
ANF9-2 M (39)	3/60	3/60	-2.0	-2.5	+	-	-	R	+	+	-	-	+	+	-	-	-	-	-	-
ANF8-1 M (12)	PL	PL	-	-	+	+	+	A	+	+	-	-	+	+	-	-	-	-	-	-
ANF8-2 M (45)	6/36	6/36	+1.5	+1.5	+	-	-	A	+	-	-	-	+	+	-	-	-	-	-	-
ANF8-3 F (8)	PL	PL	-	-	+	-	-	A	+	-	-	-	+	+	-	-	-	-	-	-

Key: PL, perception of light; CF, counting fingers; A, absent; R, remnant; NA, not applicable; 1, trabeculectomy: 2, cataract surgery

**Table 2:** Summary of genetic variation in familial aniridic patients

S No	Systematic Name	Codon	Exon/Intron	Domain	Common Name patient ID	Type of Mutation	Interpretation	Gene bank Accession No.
01	c.1080 C>T	R240X	exon 9	Homeodomain	ANF/6-1 female	Premature termination	Arginine to stop codon	--
03	c.1174del TG	271	exon 10	PST region	ANF/2-1 female	Deletion	Novel mutation	DQ251040
05	c.710del C	116	exon 6	Paired domain	ANF/7-1 male	Deletion	Novel mutation	DQ251039
07	c.406delTT	15	exon 5	Paired domain	ANF9-1female	Deletion	Novel mutation	DQ251038
09	c.393ins5	11	exon 5	Paired domain	ANF8-1 male	Insertion	Novel mutation	DQ251037

### Family ANF6: mutation c.1080C>T

**Figure 1:**
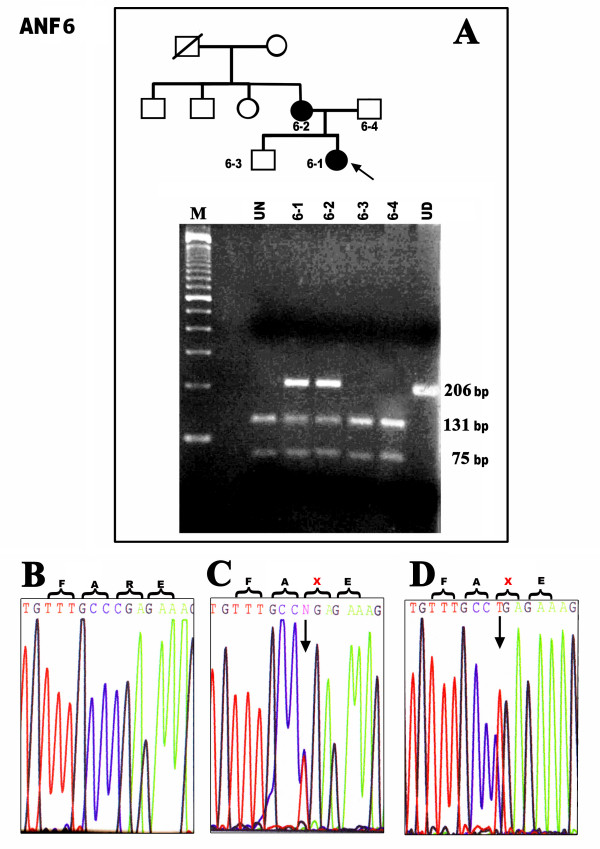
**Detection of a nonsense mutation (R240X) in the two-generation family ANF6**. **A- **Pedigree showing the affected (dark filled) and unaffected (unfilled) family members. Cloned gPCR product digested with *AvaI *restriction enzyme confirms heterozygosity for the mutation in affected individuals (band sizes 206 bp, 131 bp, 75 bp in lane 2, 3) whereas the unaffected members carry only normal alleles with digested bands at 131 bp and 75 bp (lane 1 and 4). The unrelated normal control is shown on the left (UN). Undigested PCR product is on the right (UD). **B- **Normal chromatogram of *PAX6 *shows the presence of CGG (arginine) in exon 9. **C- **Direct sequencing of the genomic PCRs of the affected mother and **(D) **affected proband showing the presence of mutation, X-represents stop codon.

The mother and her daughter were affected with aniridia in the two-generation family ANF6. Sequencing revealed a transition in exon 9, **c.1080C>T **in both affected individuals. The unaffected family members showed normal alleles of *PAX6*. The C>T substitution at codon 240 converts an arginine codon (CGA) to a termination codon (TGA) (**R240X**). The mutation was predicted to remove an *AvaI *site. A 206 bp band around the mutation was amplified and digested with *AvaI *restriction enzyme to reconfirm the presence of the mutation in exon 9 of *PAX6*. The digested product of the normal allele yielded 131 bp and 75 bp fragments on a 1.5% agarose gel in unaffected individuals whereas 206bp, 131bp, and 75bp fragments were obtained from individuals heterozygous for the mutant allele (Fig. 1A). This mutation has been reported previously in various ethnic populations but this is the first report from familial aniridia in India [5,6].

### Family ANF2: novel mutation c.1174delTG

[Genbank:DQ251040]

**Figure 2:**
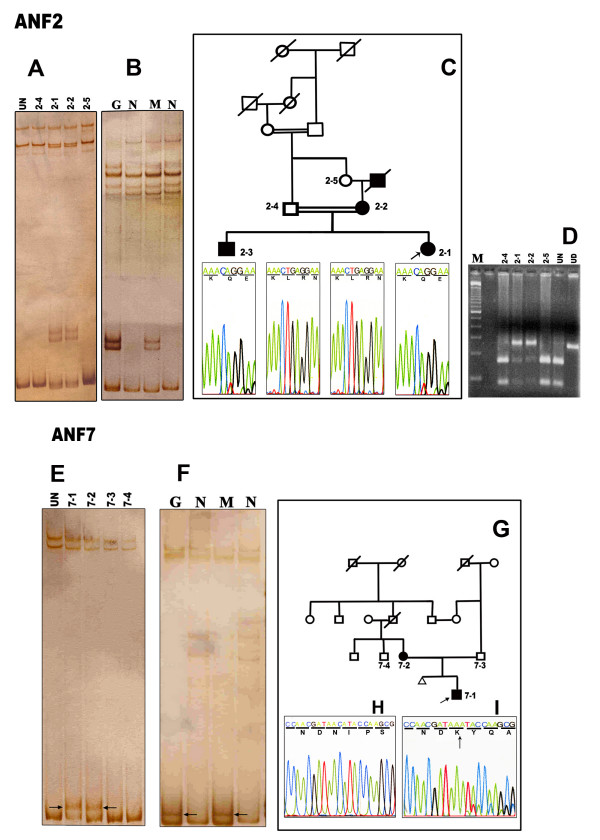
**Mutation analysis of two unrelated families, ANF2 and ANF7**. **A**. SSCP gel stained with silver nitrate showing unaffected members (2–4, 2–5) and the unusual band shift in affected members (2–1, 2–2), and normal bands in an unrelated unaffected control (lane 1). **B**. Identical banding patterns were obtained from the cloned mutant (M) and the genomic mutant (G) DNA on silver stained polyacylamide gels. N represents normal alleles. **C**. Three generation aniridic pedigree showing the affected and unaffected members and sequencing results of cloned mutant alleles showing the mutant (c.1174delTG) and wild-type alleles of exon 10. **D**. Restriction digestion analysis: PCR products of the family ANF2 showed heterozygosity (2–1, 2–2) for the mutant allele (243 bp, 160 bp and 83 bp). The wild type shows bands at 160 bp and 83 bp (2–4, 2–5, UN) when digested with *DdeI*. UN represents an unaffected control. UD represents undigested PCR product of exon 10. **E**. Polyacrylamide gel showing the bandshift in affected members (7–1, 7–2) but no shift on normal family members or unrelated controls (UN) (UN, 7–3, 7–4) of family ANF7. **F. **Allele specific clones were isolated to confirm the correct clone (M) compared with genomic PCR (G). **G**. The pedigree showing the two-generation aniridic family (filled-affected, unfilled-unaffected). **H**. Sequence of the unaffected father showing wild type *PAX6*. **I**. Sequencing of cloned alleles revealed the presence of single cytosine residue deletion in exon 6 in the mutants.

Affected individuals from the three-generation family ANF2 showed bands with altered mobility shift on SSCP gels and were shown by sequencing to be heterozygous for the mutation **c.1174delTG **at codon 271 in exon 10 (Fig. 2A, C). The affected aniridic mother passed the mutation onto two children. This deletion leads to a frameshift, alters a leucine residue (codon 271) and creates a premature termination codon 12 residues downstream. The conformations observed both in genomic DNA and the cloned PCR products showed the same profiles (Fig. 2A, B). The mutation was predicted to alter a *DdeI *site in exon 10 and the mutation was reconfirmed by *DdeI *restriction of PCR products from both in unaffected controls and in mutants. The wild-type alleles of unaffected individuals and unrelated normal controls showed two bands, 160 bp and 83 bp, whereas DNA from the affected individuals yielded 243 bp, 160 bp and 83 bp bands (Fig. 2D), confirming the loss of the *DdeI *restriction site on one allele of affected individuals in this family.

### Family ANF7: novel mutation c.710delC

[Genbank:DQ251039]

The PCR products of affected individuals from the two-generation family ANF7 (Fig. 2E) showed aberrant banding patterns on SSCP analysis. Sequencing indicated a deletion mutation **c.710delC **in all the affected individuals in this family. The conformation profiles of genomic PCR-SSCP and the cloned allele-specific PCR products showed the same pattern (Fig. 2E, F). Deletion of the single cytosine residue (Fig. 2I) is predicted to lead to a frameshift at codon 116 (asparagine) of exon 6 (Fig 2I) and lead to a stop codon in exon 7 within the paired domain. The PCR product of the unaffected father showed a wild-type allele of *PAX6 *(Fig. 2H).

### Family ANF9: novel mutation c.406delTT

[Genbank:DQ251038]

**Figure 3:**
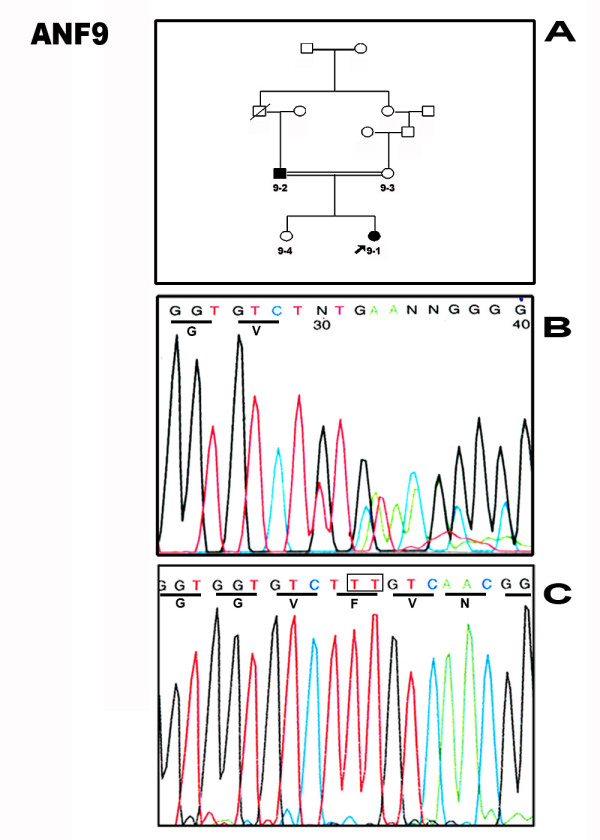
**Pedigree and the sequencing analysis of the family ANF9**. **A. **Two generation family pedigree showing the affected (filled) and unaffected members (unfilled). **B. **Direct sense strand sequencing of the genomic PCR of the affected proband showing superimposed signals revealing heterozygosity for the deletion mutation. **C. **Exon 5 of sequencing from an unaffected relative showing the presence of the two base pairs missing in the mutation (BOX).

In the two generation consanguinous family ANF9 (Fig. 3A), an obvious bandshift on the SSCP gel was seen which, on sequencing, proved to be due a two base pair deletion at **c.406delTT **that disrupts a phenylalanine at codon 15 in the paired domain (Fig. 3B). The mutation resulted in frameshift in exon 5 and creates the stop codon at exon 6 (40 codons distal to the mutation). Normal alleles were found in unaffected family members (Fig 3C).

### Family ANF8: novel mutation c.393ins5

[Genbank:DQ251037]

**Figure 4:**
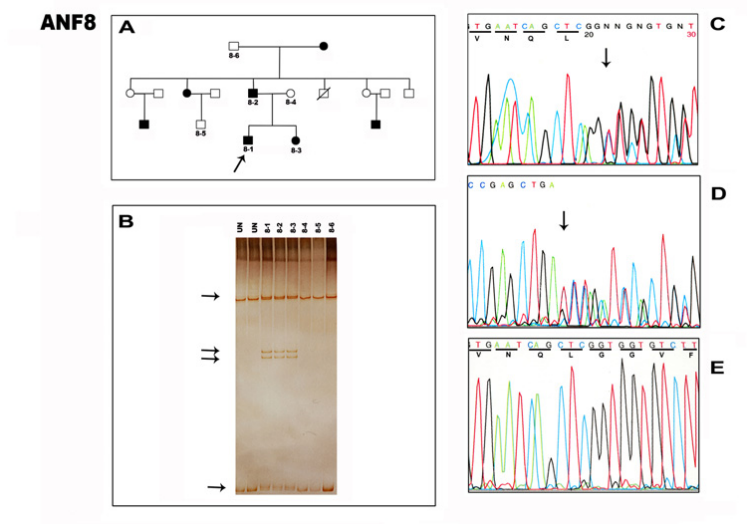
**Detection of five base pair duplication mutation in exon 5 of PAX6 in family ANF8. A. **Three generation pedigree shows the large family with affected and unaffected members. Arrow denotes proband. **B. **SSCP gel shows the unusual banding pattern (arrow) in all the affected members (8–1, 8–2, 8–3) but not in unaffected members or unrelated controls (UN) **C. **Forward and (**D**) reverse sequencing of genomic PCRs showing the superimposed signal distal to the duplication (arrow). A sequencing artifact is visible to the left of this trace, but the final edited data sequence is presented from several replicates of forward and reverse sequence. **E. **Forward sequencing result of normal exon 5 of *PAX6 *from the unaffected family member.

The mutation in another three generation family ANF8 was originally detected by SSCP in exon 5 (Fig. 4A, B) of the paired domain and was further analyzed by sequencing the PCR product of affected individuals and unaffected relatives from the family. The DNA sequence of the PCR product of affected heterozygous individuals showed a 5 base pair insertion duplication (**TCAGC**) at **c.393ins5 **(codon 11) (Fig 4C, D). This results in a frameshift mutation at a leucine residue, which produces a premature termination codon 20 codons distal to the mutation location, in exon 5 within the paired domain. The male ANF8-1 had sudden loss of vision at age 11 and anterior segment examination revealed bilateral microcornea with aniridic keratopathy. Another affected 8-year old female ANF8-3 also had microcornea with post polar cataract in the right eye.

Five mutations in the coding regions have therefore been documented in the family study, in which we have identified four novel mutant *PAX6 *alleles in Indian familial aniridic patients.

## Discussion

Most familial aniridia patients have mutations within *PAX6 *that are dominantly inherited with high penetrance. Sporadic cases are likely to have *de novo *mutations in *PAX6*, and expected to be transmitted to the next generation in an autosomal dominant fashion. In this study, we observed several novel *PAX6 *mutations in familial aniridia. Affected family members in at least two generations carry the mutant alleles.

SSCP is an easy, but perhaps inefficient method to screen for mutations in genomic DNA. In this study, five mutations were found in *PAX6 *in affected members from nine unrelated families with inherited aniridia. All the mutations were identified initially based on aberrant bands on SSCP gels that differed from the normal allelic patterns shown by unaffected family members. Each mutation was only found in affected individuals.

Many mutations in PAX6 generating premature termination codons have been previously reported [10] and are deposited in the Human *PAX6 *Allelic Variant Database [9]. A c.1080C>T change (R240X), identical to that in our family ANF6, that was identified recently in a Thai study also showed complete aniridia with nystagmus, strabismus and foveal hypoplasia [10] c1080C>T has been reported several times previously in other ethnic groups and appears to represent a hotspot of PAX6 mutation, perhaps due to methylated cytosine deamination. The c.393insTCAGC insertion in ANF8-1 is novel, but a c.395delC mutation with familial aniridia was previously reported which lies in same codon of *PAX6 *but two base pairs down [12].

The mutant mRNAs predicted by this study are likely to be detected by RNA surveillance and degraded by nonsense-mediated decay [13,14], and hence we predict that they will all represent loss-of-function mutations.

## Conclusion

Our genetic analysis provides further examples of *PAX6 *haploinsuffiency leading to aniridia. We report four novel frameshift mutations and one nonsense mutation in Indian aniridic pedigrees. This is the first genetic analysis of familial aniridia in Indian populations and contributes to our understanding of the relationship between *PAX6 *genotype and ocular phenotype.

## Competing interests

The author(s) declare that they have no competing interests.

## Authors' contributions

GN carried out the molecular genetic analysis. JN assisted in techniques. SRK, PV, and SS contributed for the clinical diagnosis of patients. PS conceived the study carried out the molecular analysis and drafted the manuscript with GN. JMC critically reviewed the study, provided intellectual input, revised and formatted the manuscript. JMC drafted the final version of the manuscript.

## Pre-publication history

The pre-publication history for this paper can be accessed here:


